# 

**Published:** 1887-09

**Authors:** 


CONTENTS:

ARTICLE I.—Teething: Is it a Common Cause of Disorder. By Selwyn A. Russell, M. D .......... 193

" II.—Management of Children's Teeth. By J. E. Cravens, D. D. S .......... 206

" III.—Educated Ability and Industry Essential to Success in the Practice of Dentistry as a Specialty. By J. H. Grant .......... 209

" IV.—Anæsthetics and Anæsthesia. By J. L. Fountain .......... 218

" V.—Resolution of American Medical Association .......... 225

" VI.—Florida Dental Law .......... 227

" VII.—Gold at Cervical Border. By Wm. H. Cooke .......... 229

EDITORIAL, ETC.

Southern Dental Association .......... 231

The International Medical Congress .......... 232

Death in a Dental Office .......... 232

MONTHLY SUMMARY.

Toxic Effects of Lead .......... 235

One Objection to Plastic Fillings .......... 235

The American Medical Association Recognizes Dentistry as a Specialty in Medicine .......... 236

Treatment of Diarrhœa by Iodoform and Charcoal .......... 237

Hypnotic Experiments in Paris .......... 238

Spiders .......... 239

A Case of Replantation .......... 239

Myrtol .......... 240

The Adulteration of Candies .......... 240

Pulse in Morphiomania .......... 240

